# Effect of high temperature on mechanical properties of lithium slag concrete

**DOI:** 10.1038/s41598-024-62837-y

**Published:** 2024-05-24

**Authors:** Jiongfeng Liang, Wanjie Zou, Yongliang Tian, Caisen Wang, Wei Li

**Affiliations:** 1 Faculty of Civil & Architecture Engineering, East China University of Technology, Nanchang, China; 2 College of Civil and Architecture Engineering, Guangxi University of Science and Technology, Liuzhou, China; 3 College of Civil and Architecture Engineering, Wenzhou University, Wenzhou, China; 4 Key Laboratory of Engineering and Technology for Soft Soil Foundation and Tideland Reclamation of Zhejiang Province, Wenzhou, China; 5 Faculty of Architecture, Civil and Transportation Engineering, Beijing University of Technology, Beijing, China

**Keywords:** High temperature, Mechanical properties, Lithium slag, Supplementary cementitious material, Green concrete, Engineering, Materials science

## Abstract

As the main gel material of concrete, cement is used in an astonishing amount every year in the construction industry. However, a large amount of CO_2_ is emitted into the atmosphere while producing cement. Therefore, it is the general trend to look for substitutes for cement and develop new green concrete. Lithium slag (LS) is the industrial waste discharged from lithium salt plants. Through testing, it is found that the chemical composition of LS has a high degree of coincidence with ordinary Portland cement (OPC) Therefore, LS can be incorporated into concrete as supplementary cementations material (SCM) to prepare lithium slag concrete (LSC). The pollution of the natural environment caused by a large number of piled-up and landfilled LS is immeasurable. Consuming and using LS in large quantities and with high efficiency not only eliminates the pollution of lithium slag to the natural environment, but also helps to reduce the amount of cement used in green concrete and truly reuse waste resources. In order to study the mechanical properties of post-heated LSC, the test were carried out for LSC specimens after high-temperature. The main influence factors were considered, including the temperatures of 20℃, 100 ℃, 300 ℃, 500 ℃ and 700 ℃, the contents of lithium slag in LSC of 0%, 10%, 20% and 30%, cooling method of LSC after exposure high temperature. The results showed that the mechanical properties of LS concrete specimens were slightly improved at 100 ℃, and when the temperature was 300 ℃ or higher, the damage to the specimens was huge and irreversible. An appropriate amount of LS (20% lithium slag content) could improve the strength of LSC. This paper also studied the relationship between lithium slag content and strengths of LS concrete. The research results show that adding an appropriate amount of LS to concrete improves the mechanical properties of concrete. When the LS replacement rate is 20%, the mass loss rate of LSC after different high temperature treatments was the minimum. The cubic compressive strength, axial compressive strength, and flexural strength of specimens with 20% LS substitution can be increased by 8.16%, 8.33%, and 13.46% after high temperature. The cubic compressive strength, axial compressive strength, and flexural strength of specimens with 20% LS substitution can be increased by 8.16%, 8.33%, and 13.46% after high temperature.

## Introduction

Concrete is one of the most widely used construction materials, and the global demand for concrete is as high as 11.5 billion tons per annum^1,2^. The output of concrete is more than 10 times that of steel worldwide^3^. Globally, approximately 2 billion tons of cement are produced annually^4^. According to the survey, 0.9 tons of carbon dioxide are emitted for every ton of cement produced^5,6^. On a global scale, the CO_2_ gas emitted to nature due to the production of cement accounts for 8% of the total CO_2_ emissions^4,7^. As a non-sustainable material, traditional concrete is used in such a large amount that it violates the principles of sustainable development and environmental protection in modern society^8^.

The concept of green concrete^9^ was proposed by Boobalan et al. in 1998, which not only had the excellent characteristics of concrete, but also could reduce the damage to the environment, and could even use and consume waste materials that pollute the environment. Once such a concrete with broad application prospects was proposed, a large number of scholars poured into this field. By adding distinctive types of materials to the concrete, it studied the mechanical properties of the new concrete, hoping to find suitable materials for the concrete, and transform the green concrete from a conceptual model into a real commodity for use in actual projects.

As a powdered water-hardening inorganic cementitious material, cementitious materials play the role of “binder” in concrete. Due to the pollution of the environment during the production and use of cement, many scholars have begun to look for alternative materials to cement, supplementary cementitious materials (SCM) also known as mineral admixtures^10–14^. The industrial production process generates a large amount of solid waste, i.e. industrial by-products, which are poorly utilized and generally disposed of in piles and landfills, which are particularly polluting to nature, especially groundwater.

Lithium slag (LS) is an unutilized industrial by-product in the production of lithium salts. Since the 1970s, the market demand for lithium has increased by more than three times^15,16^, and the consumption of lithium products continues to rise. Studies have shown that for every 1 ton of lithium salt produced, about 9 tons of LS is discharged^17^. The preparation of lithium slag concrete (LSC) by adding lithium slag to concrete not only consumes a large amount of lithium slag and reduces the amount of cement, but also improves the properties of concrete.

He et al.^18^ replaced cement with LS in equal amounts (0%, 10%, 20% and 30%) to study the effect of LS content on the mechanical properties of concrete. The results showed that LSC with 20% LS content had the highest compressive strength after curing for 60 and 90 days. In addition, 20% LS content can reduce the drying shrinkage strain to the greatest extent. Zhang et al.^19^ tested the acid rain corrosion resistance of LSC. The results showed that LS can accelerate the hydration reaction of concrete, and 40% LS content has the best effect on improving the acid rain resistance of LSC. Zhai et al.^20^ observed the microstructure of lithium slag cement paste at different hydration ages and found that when the LS content was 10–30%, the internal structure of lithium slag cement paste after curing for 28 days was more compact. In addition, the experimental results showed that the Ca(OH)_2_ content in lithium slag cement paste was lower than that in pure cement paste, proving that LS has strong activity. Rahman et al.^21^ found that after 28 days of curing, the compressive strength of LSC with 20% LS content was the highest (49.3 MPa), while the compressive strength of LSC with 40% LS content was the highest (58.6 MPa) after 90 days of curing. This indicates that a larger amount of LS (40%) in LSC will continue to improve its strength. After long-term curing, LSC will have better mechanical properties. Amin et al.^22^ found that LSC with LS content of 20–40% had greater compressive strength, reduced permeable voids, water penetration, sorptivity and porosity, and its transport properties were greatly improved. In summary, when LS replaces cement in medium-quality concrete at a ratio not exceeding 40%, it can not only improve the mechanical properties of concrete but also reduce the use of cement, indicating that the use of LSC in future projects is promising.

The global annual loss of buildings due to fire is huge, although concrete has good fire resistance and is a non-combustible material. However, when exposed to open fire or high temperature environment for a long time, the mechanical properties will be weakened to different degrees. Therefore, it is extremely important to study the effect of high temperature environment on the damage and residual strength of different concretes^23–26^. It should be noted that the natural cooling (NC) after high temperature exposure is significantly different from the cooling method of concrete buildings in the event of an actual fire, and water spray is mainly used to extinguish the fire in the event of a fire. Many literatures have concluded that water spray cooling (WSC) will lead to a serious decrease in concrete strength^27–29^.

For buildings made of LSC as the main material, the fire resistance and high temperature resistance of the building itself are particularly important. Therefore, it is particularly important to conduct a comprehensive and systematic study on the mechanical properties of LSC after high temperature. However, after consulting a large number of literatures, it was found that the research on the mechanical properties of LSC after high temperature is still lacking. Therefore, in this paper, the mechanical properties of LSC were tested and analyzed. The effect of the heating temperature, the lithium slag content of LSC and the cooling method on the compressive strength, prism compressive strength and flexural strength of LSC was studied. Through the data obtained from the test, the functional relationship between temperature and the strength of LSC is proposed, which can not only predict the loss of strength of LSC at different temperatures, but also provide data support for the repair and reinforcement of LSC buildings after actual fire.

## Experiment

### Raw materials

**Figure 1:**
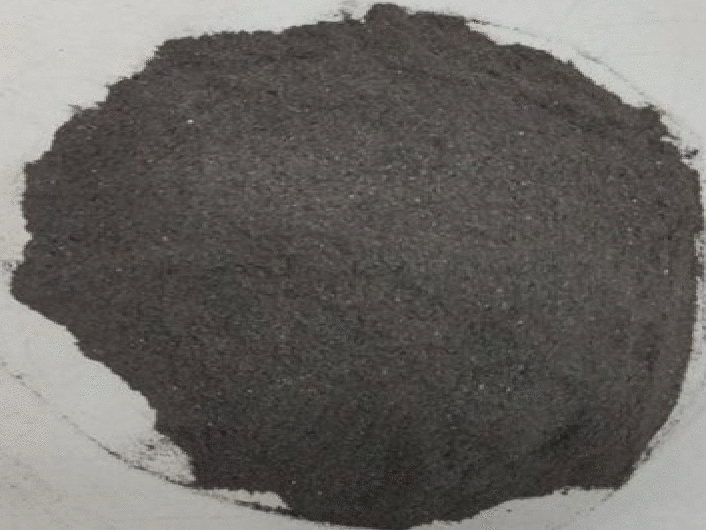
Appearance of LS.

PO 32.5 ordinary Portland cement (OPC) was used in this test, and LS used came from Xinyu Lithium Salt Factory in Jiangxi Province, China. The appearance color of LS was purple-brown, as shown in Fig. 1.

**Figure 2:**
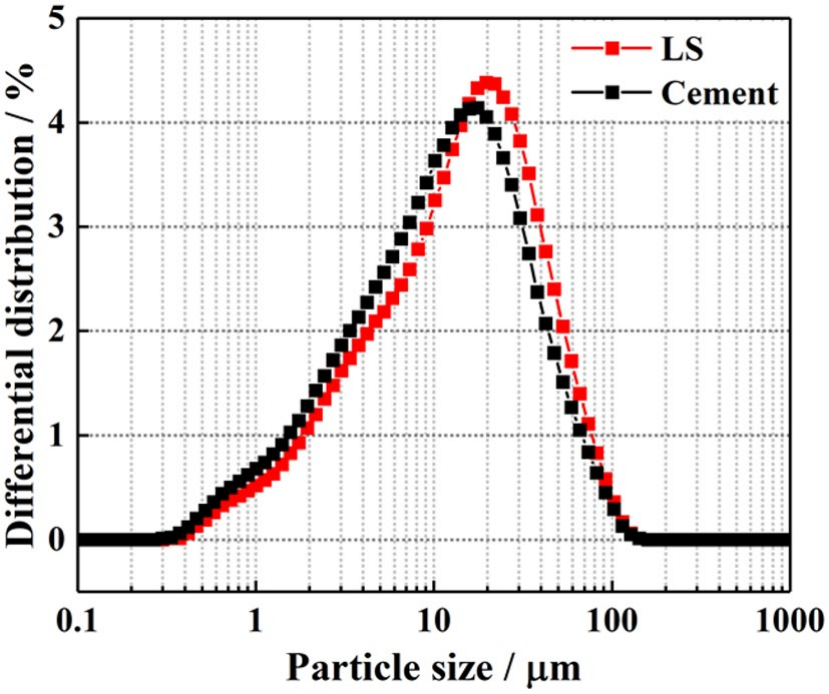
Particle size distributions.

**Figure 3:**
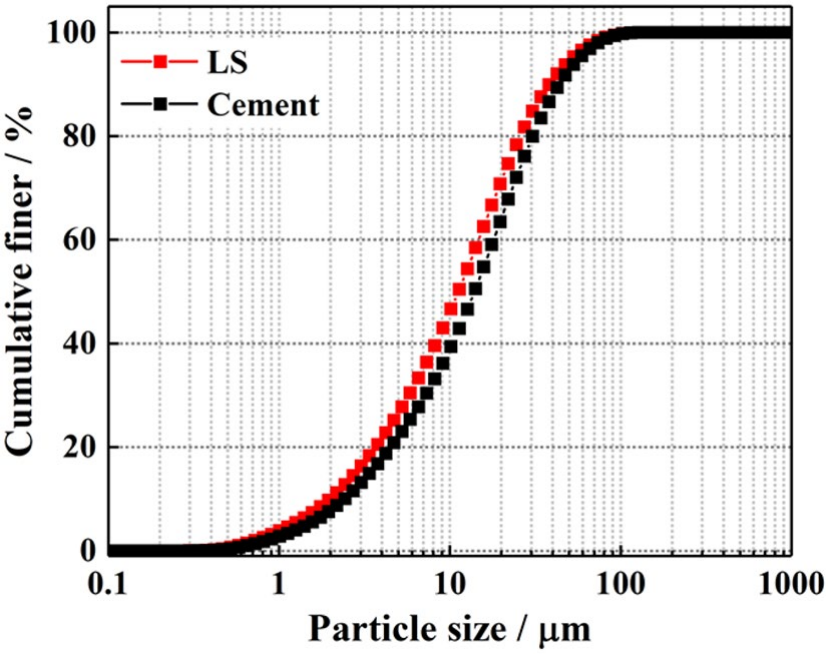
Gradation curve.

Laser particle analysis of LS was carried out with BT-9300LD laser particle size distribution analyzer, and the particle size distribution and gradation curve of OPC and LS were measured. It can be seen from Figs. 2 and 3 that the particle size of LS was very close to that of OPC, and the average particle size of LS was smaller.

**Figure 4:**
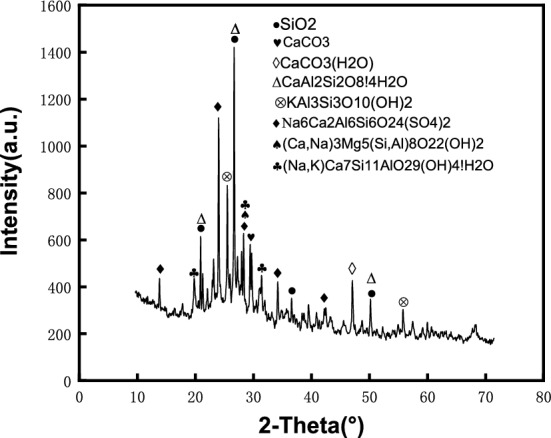
XRD pattern of the LS.

The LS was detected with an X-ray diffraction (XRD), and the mineral composition of the LS was measured as shown in Fig. 4. The detailed chemical compositions of cement and LS are shown in Table 1. Comparing the two sets of data in Table 1, it can be found that the chemical composition types of the two were highly similar. The composition of SiO_2_ and Al_2_O_3_ in LS was more than that of OPC, but the content of CaO in LS was much less than that of OPC. In addition, LS had more SO_3_ content than OPC.

**Table 1:** Chemical compositions (wt%).

Materials	$Si{O}_{2}$	$A{l}_{2}{O}_{3}$	$F{e}_{2}{O}_{3}$	$CaO$	$MgO$	$S{O}_{3}$	Loss on ignition
OPC	18.9	5.4	4.1	60.4	1.9	2.6	6.7
LS	57.3	17.1	1.3	10.3	0.7	3.1	10.2

As fine aggregate, river sand had a fineness modulus of 1.9, a bulk density of 1505kg/m^3^ and a compact density of 1695 kg/m^3^. Crushed stone was used as the coarse aggregate, the 30 min water absorption rate was 1.1%, the crushing index value was 6.3%, and the size range of crushed stone was 5.0 mm to 25 mm. The water used in the whole process of this experiment was city tap water.

### Mix proportions

The concrete mix ratio was confirmed according to the “*Specification for Mix Proportion Design of Ordinary Concrete*” (Chinese JGJ 55-2011)^30^. The design strength of the concrete was 30MPa.

Concrete with four LS contents (0%, 10%, 20% and 30%) for replacing cement (C) were designed for the experiment. Table 2 details the mix proportions of concrete. The water-binder ratio (W/B) of all samples was 0.45.

**Table 2:** Mix proportions (kg/m^3^).

Types	Replacement rate (%)	OPC	LS	River sand	Gravel	Water
C	0	456	0	522	1217	205
C + 10LS	10	410.4	45.6	522	1217	205
C + 20LS	20	364.8	91.2	522	1217	205
C + 30LS	30	319.2	136.8	522	1217	205

### Mixtures and specimen preparation

A total of 252 concrete specimens were tested. The cubic compressive strength, prism compressive strength and flexural strength of concrete was confirmed by three 100 mm × 100 mm × 100 mm specimens, 100 mm × 100 mm × 300 mm specimens and 100 mm × 100 mm × 400 mm specimens, respectively.

Pour the LSC mixture into a mold of corresponding size, vibrate compactly, smooth the surface with a spatula, and let it stand for 24 h. After the specimens solidified and formed, it was denuded and numbered. All specimens were put into the water tank and kept 28 days until testing.

### Heating and cooling regime

The specimens had a high moisture content due to their preservation in water. Therefore, put the specimen in an indoor dry environment to dry for several days, and weigh the mass of the specimen after the moisture content reached a normal state.

**Figure 5:**
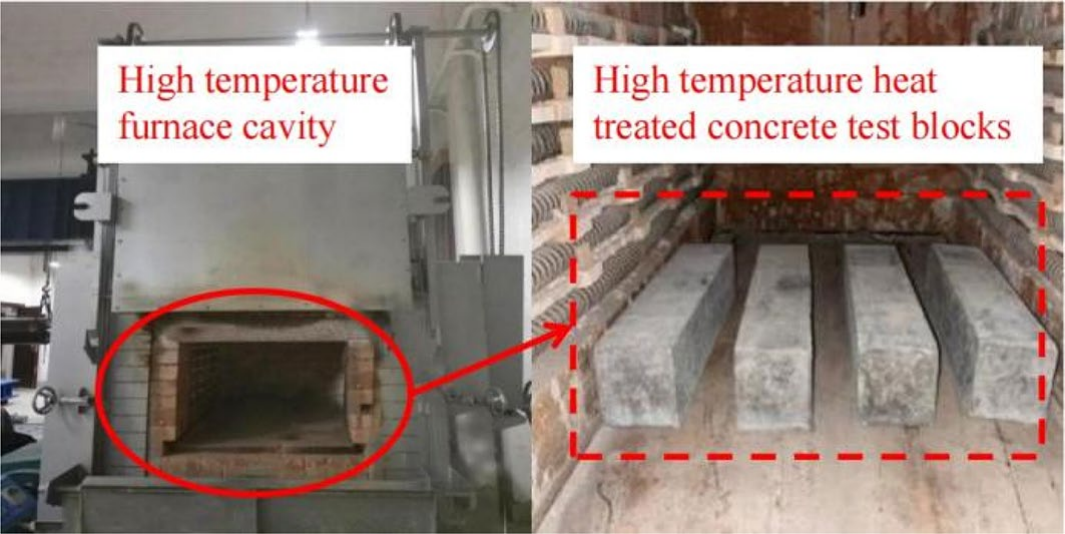
RX3-45-9 box type resistance furnace.

In an actual fire scene, according to the ISO 834 standard fire temperature rise curve^31^, the fire scene temperature will reach 700 ℃ within 30 min. As the combustibles in the fire scene decrease, the fire scene temperature will not continue to rise to above 1000 ℃, but will remain in a certain temperature range for several hours. If there are no fires extinguishing measures in the fire scenes, the fire scene temperature will gradually drop to room temperature after the combustible materials are completely burned out. Therefore, the temperature range designed in this paper is 20–700 ℃. The heating device was RX3-45-9 box-type resistance furnace, as shown in Fig. 5.

**Figure 6:**
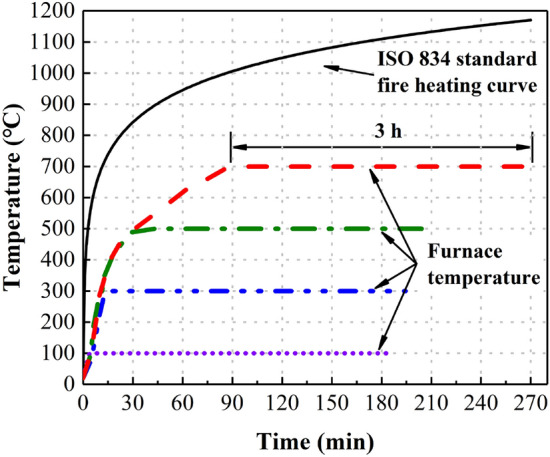
Heating curve.

Considering that the concrete specimens might explode and peel off under high-temperature environment, the specimens should be preheated before starting the high-temperature test. The duration of high temperature was three hours. Figure 6 shows the heating curve of the high-temperature test in detail.

Cooling methods for concrete specimens include natural cooling (NC) and water spray cooling (WSC). The water used in WSC is laboratory water at room temperature. After the high-temperature heating with a target temperature was completed, the furnace door can be directly opened for NC. The specimen treated by WSC need to open the furnace door quickly after the heating was finished, took out the specimens and then closed the furnace door. Because when the temperature in the high-temperature furnace was too high, the convection between the gas in the furnace and the air at room temperature would cause cracks inside the high-temperature furnace^32^.

After the high temperature test, the concrete specimens were cooled to room temperature and weighed. Since the surface of the WSC specimens absorbed a lot of water, which need to be placed in a dry environment. After the water on the surface of the specimens evaporated naturally, which were weighed and recorded.

### Test equipment and test method

In this experiment, the mechanical properties of LSC specimens were tested in accordance with the Standard for Test Methods of GB/T 50081-2019^33^. TYA-3000 electro-hydraulic press machine was used for this experimental. Table3 details the numbers of different groups of LSCs. Lithium slag replacement rate, temperature and cooling method were all determined according to the test scheme in Table 3. The control group of this test was the specimen with 0% lithium slag content under normal temperature conditions.

**Table 3:** Concrete test samples number.

Serial number			
R0-T20-NC	R10-T20-NC	R20-T20-NC	R30-T20-NC
R0-T100-NC	R10-T100-NC	R20-T100-NC	R30-T100-NC
R0-T300-NC	R10-T300-NC	R20-T300-NC	R30-T300-NC
R0-T500-NC	R10-T500-NC	R20-T500-NC	R30-T500-NC
R0-T700-NC	R10-T700-NC	R20-T700-NC	R30-T700-NC
R0-T100-WSC	R0-T300-WSC	R0-T500-WSC	R0-T700-WSC
R20-T100-WSC	R20-T300-WSC	R20-T500-WSC	R20-T700-WSC

R0, R10, R20, and R30 represent lithium slag replacement rates of 0%, 10%, 20%, and 30%, respectively; T20, T100, T300, T500, and T700 represent heating temperatures of room temperature, 100 ℃, 300 ℃, 500 ℃, and 700 ℃, respectively; *NC* represents natural cooling, and *WSC* represents water spray cooling.

## Experimental results and discussions

### Appearance

**Figure 7:**
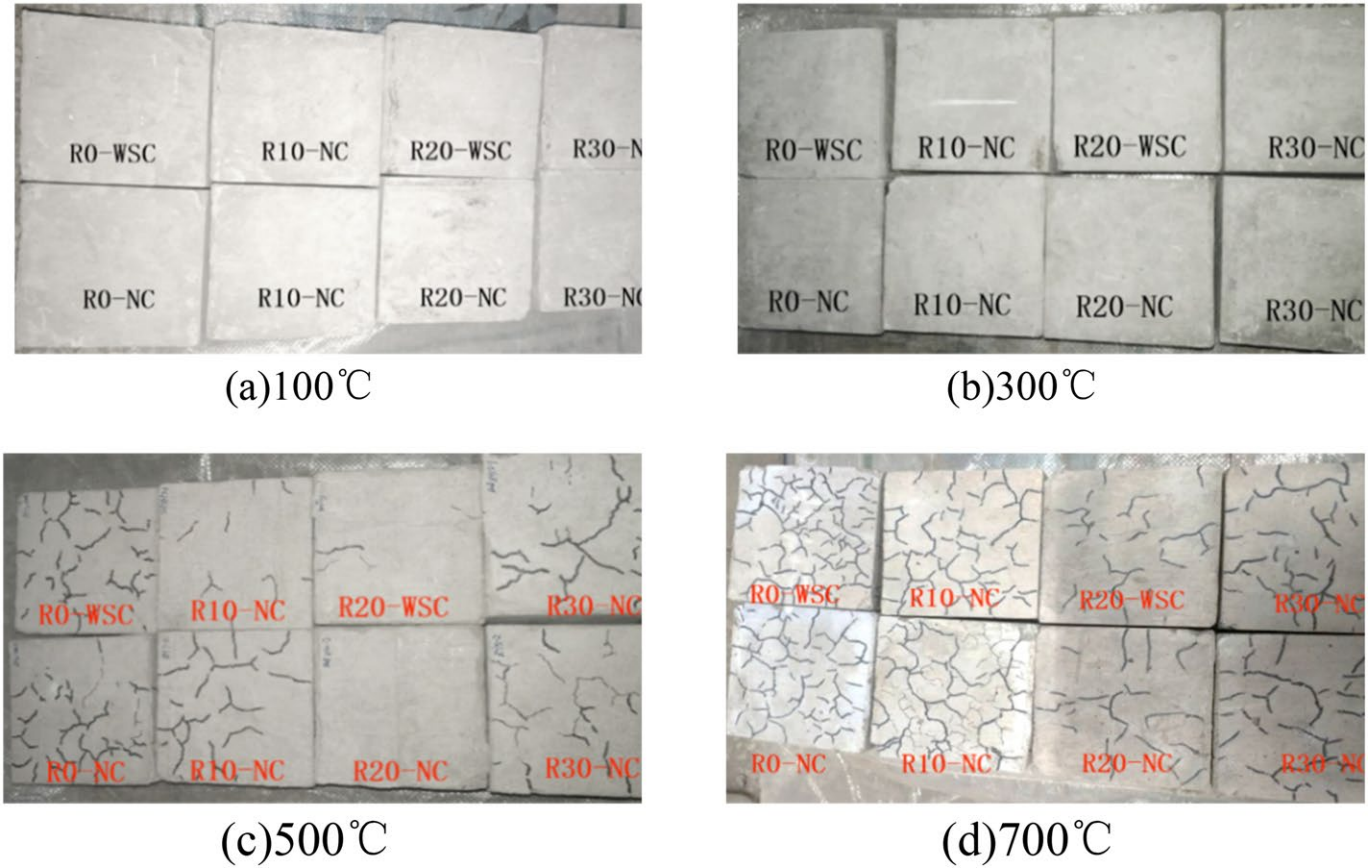
Appearance of LSC. (**a**) 100 ℃, (**b**) 300 ℃, (**c**) 500 ℃, (**d**) 700 ℃.

The appearance of the LSC specimens at different temperatures is shown in Fig. 7. The surface color of the un-heating specimen was blue-gray. After the high temperature of 100 ℃ and 300 ℃ lasted for 3 h, the color turned back to off-white without obvious cracks. After exposure to 500 ℃, the color of the specimen became pinkish white, and the appearance began to appear cracks, and the outer surface of LSC specimens with 20% lithium slag content had the least cracks. After the specimen experienced a high temperature of 700 ℃, the surface appeared brick red, the appearance of cracks increased significantly, and peeling debris appeared on the surface. This debris was due to the loss of water vapor, which led to complete dehydration of the compounds on the surface of the specimen, resulting in scaling and dusting of the mortar^34^.

Comparing the appearance of WSC and NC specimens in Fig. 7, the appearance of LSC specimens had no significant effect at 100 ℃ and 300 ℃, and the cracks on the surface of specimens cooled by water spray at 500 ℃ and 700 ℃.

### Failure mode

**Figure 8:**
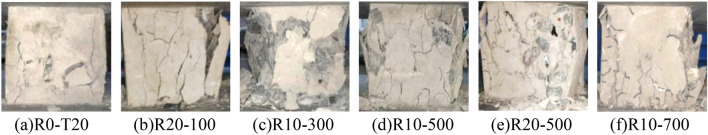
Failure modes of LSC cube specimens. (**a**) R0-T20, (**b**) R20-100, (**c**) R10-300, (**d**) R10-500, (**e**) R20-500, (**f**) R10-700.

Figure 8 shows compression failure form of the cubic concrete specimen. It showed that the failure patterns of the cubic specimens of ordinary concrete and LSC were similar. The load began to increase, and debris peeled off at the upper of the specimen, and longitudinal cracks appeared after loading to 80% of the ultimate load. Cubic specimens treated at 500 ℃ and 700 ℃ had a certain number of cracks on the surface before the loading, as the load was gradually applied to the specimens, the existing cracks on the specimen surface were further extended and widened, and new cracks were formed. After the load reached the peak value, the cracks extended into the interior of the specimen and appeared obvious cracks, large aggregates fell, and the bearing capacity decreased. When the bearing capacity of the specimen dropped to 85% of the peak value, the specimen peeled off in a large area, the load dropped rapidly, and finally the specimen was crushed.

**Figure 9:**
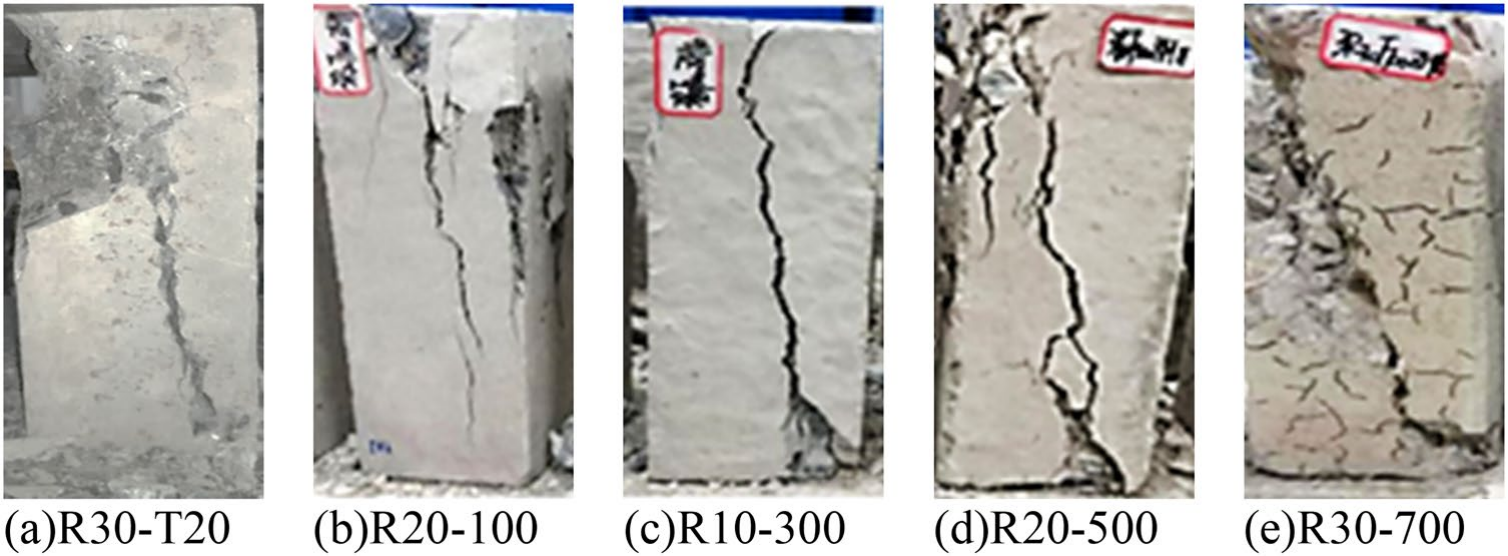
Failure modes of LSC prism specimens. (**a**) R30-T20, (**b**) R20-100, (**c**) R10-300, (**d**) R20-500, (**e**) R30-700.

**Figure 10:**
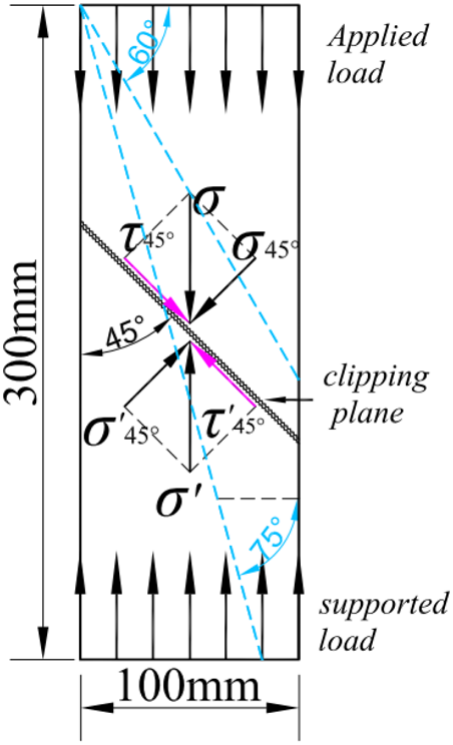
Schematic diagram of crack formation for prism concrete specimen.

Figure 9 shows the compression failure morphology of the prism specimen. It showed that the compression failure patterns of ordinary concrete and LSC were similar. The angle between the oblique failure plane and the axis after the failure of the specimen under compression was between 60° and 75°. The reason was that under the action of axial compressive stress, the maximum shear stress appeared in a direction 45° from the compressive stress, causing the specimen to be "sheared" obliquely, which showed in Fig. 10.

**Figure 11:**
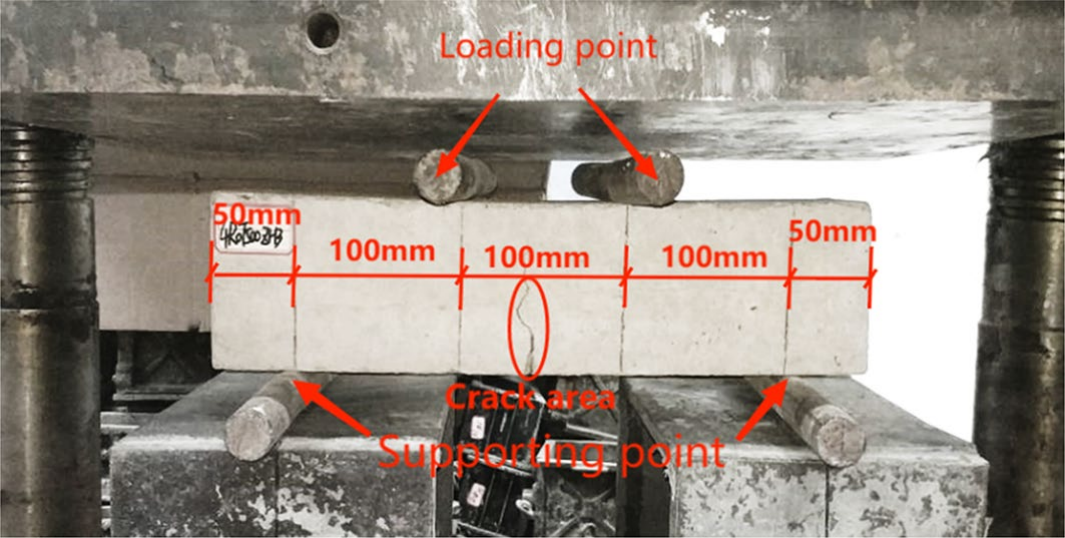
The flexural test of the prism specimen.

Figure 11 shows the flexural test of the prism specimen. The fracture section was mainly broken by gravel, which directly led to a rapid decrease in the bearing capacity of the prism specimen. Before the fracturing of the specimen, no cracks appeared on the surface, and the fracture was suddenly, showing obvious brittle mechanical characteristics. The fracture sections of the prisms were all within the range of the 100mm line drawn in the middle of the specimen, which met the requirements of GB/T 50081-2019^33^.

### Mass loss rate (ML)

**Figure 12:**
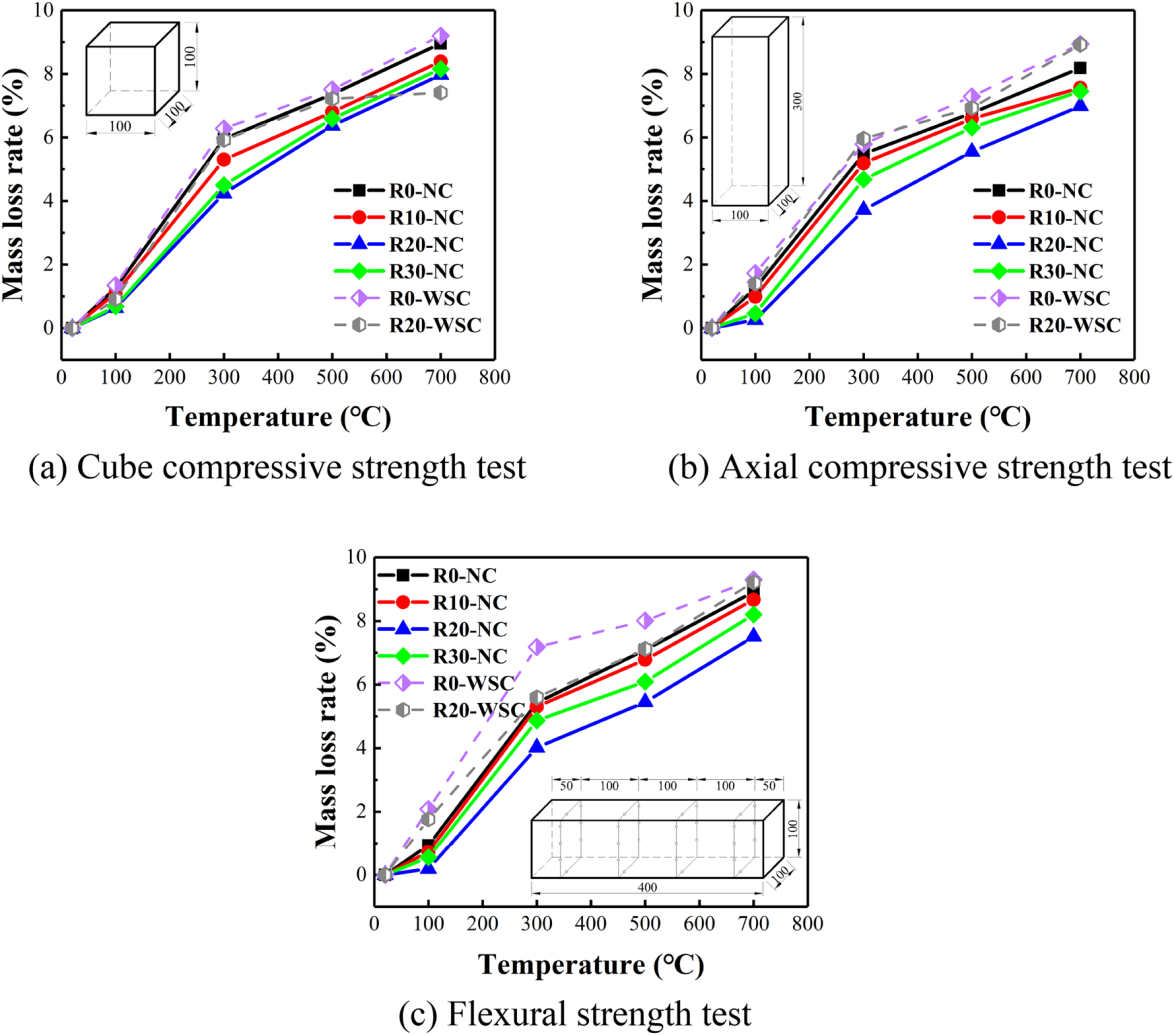
ML of the concrete specimens. (**a**) Cube compressive strength test, (**b**) Axial compressive strength test, (**c**) Flexural strength test.

The mass loss of LSC specimens after high temperature is shown in Fig. 12. When the temperature was 100 ℃, the mass loss of LSC specimens was very little. The reason was that the specimens was continuously heated at 100 ℃ for 3 h, and the mass loss was mainly due to the evaporation of free water on the surface of the sample, while the internal pores of the sample free water and bound water would not be greatly affected. Scholar Okpalaet et al.^35^ placed the concrete specimens in an environment of 105 ± 1 ℃ for drying treatment to prevent explosive spalling. Gallé et al.^36^ suggested a drying temperature of 90 ℃, and it believed that 105 ℃ would affect the microstructure of concrete. The above-mentioned two scholars regard a temperature close to 100 ℃ as the drying temperature, which could prove that 100 ℃ had little effect on concrete. In the process of increasing the temperature from 300 to 700 ℃, the mass loss of concrete specimens continued to increase, and there was a linear trend between the two.

The existing research found that the evaporation of free water in concrete was the main cause of mass loss, and increasing the density of concrete would reduce mass loss^34^. After the same high-temperature, the mass loss of LSC specimens with 20% LS substitution rate was the least. The content of SiO_2_ in LS was high (57.3%), and the content of CaO in OPC was high (60.4%). CaO and H_2_O reacted to form calcium hydroxide (CH). SiO_2_ and CH were the basic substances for pozzolanic reactions. A large amount of SiO_2_ and CaO generated more C–S–H with the help of H_2_O, so that the concrete microstructure was denser, and the water loss in LSC would be reduced.

When the lithium slag content is 10%, the mass loss of lithium slag concrete after high temperature has been reduced to a certain extent. This is because the large amount of silica in the lithium slag promotes the volcanic ash reaction of the concrete, making the internal microstructure of the concrete dense, filling many tiny gaps, and effectively inhibiting the escape of free water in the concrete. When the lithium slag content is increased to 20%, the mass loss of lithium slag concrete after high temperature is minimized. This indicates that in concrete, when lithium slag replaces cement at a ratio of 20% by mass, the volcanic ash reaction of lithium slag concrete during the curing and hardening process is the most complete. The silicon dioxide in the lithium slag reacts with the calcium oxide in the cement under the action of water to the greatest extent to produce a large amount of CSH, making the interior of the concrete more dense, making it most difficult for the free water in the lithium slag concrete to escape after high temperature. When the lithium slag content is increased to 30% again, the mass loss of lithium slag concrete after high temperature is greater than that of concrete with 20% lithium slag content. This phenomenon occurs because excessive addition of lithium slag into concrete inhibits the volcanic ash reaction of lithium slag concrete, and the excessive lithium slag mainly plays a filling role in concrete.

At 100℃, spraying water would make a small amount of water seep into the concrete, replenishing the water evaporated in the concrete due to the high temperature. When the temperature arrived at 300 ℃, 500 ℃ and 700 ℃ respectively. The mass loss of concrete specimens was composed of two parts^37^. One was that the free water in the capillary pores of the concrete specimen evaporates, and the other was that the dust and debris peeled off the surface of the concrete specimen were washed away during the spraying process, thereby reducing the mass of the specimen.

### Cube compressive strength

**Figure 13:**
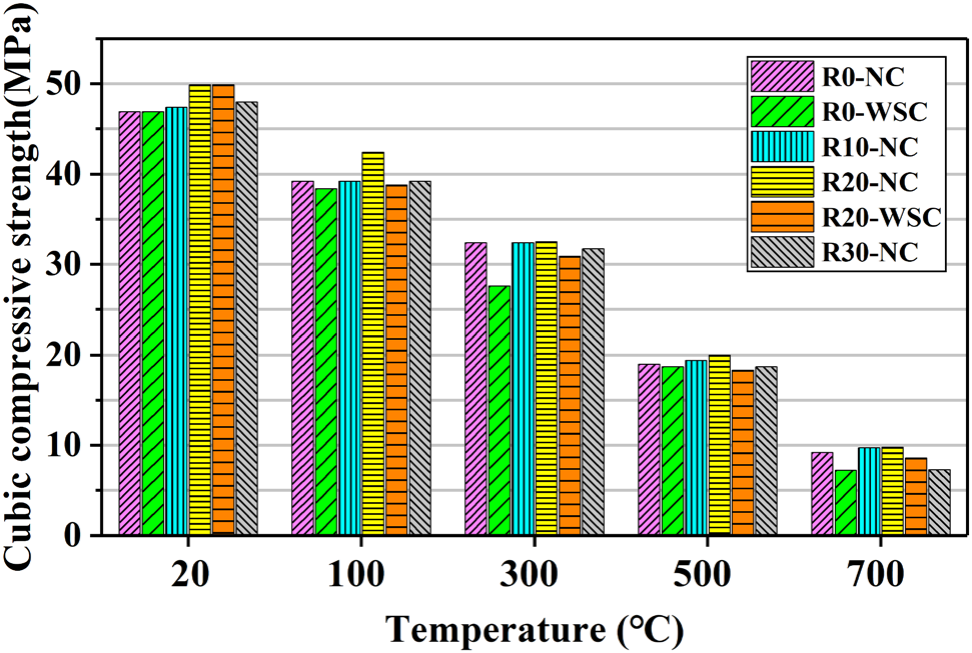
Cubic compressive strength of LSC.

**Figure 14:**
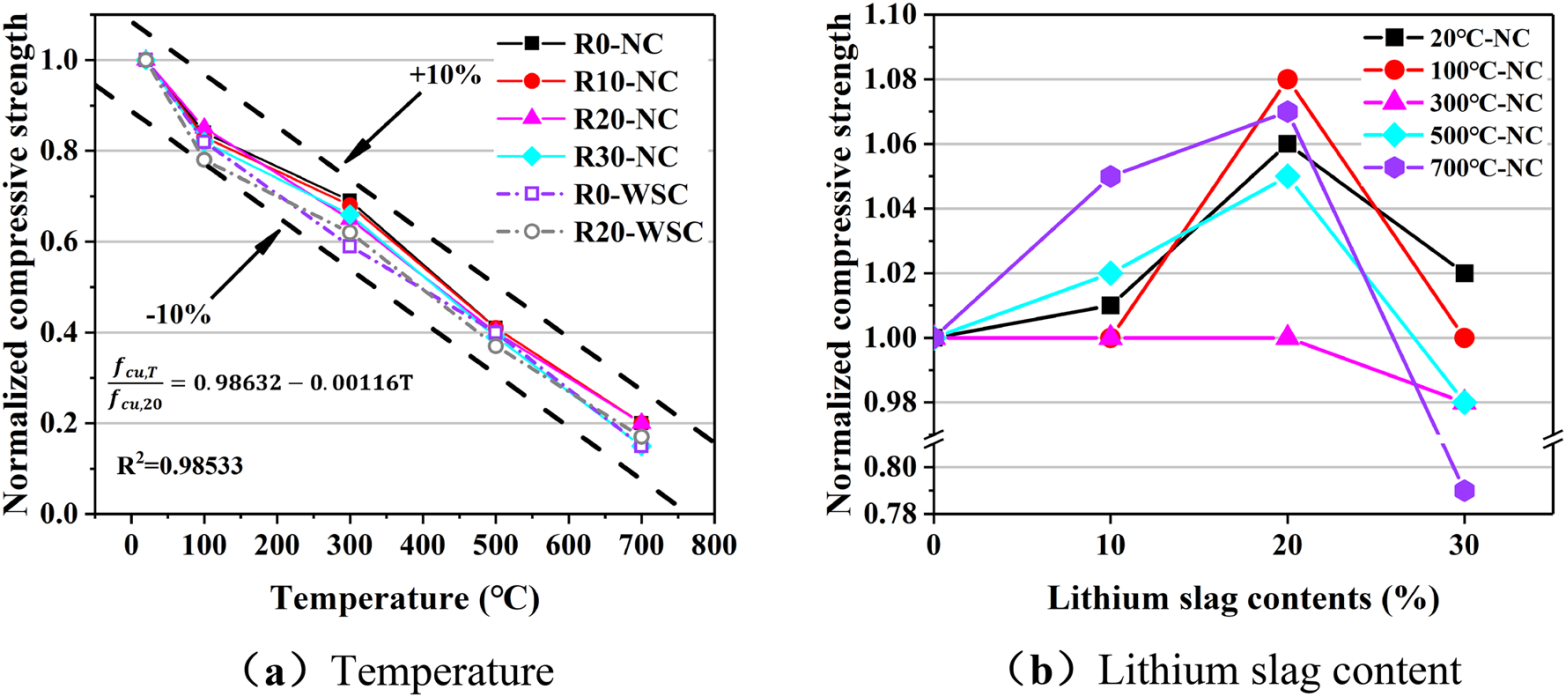
Normalized cubic compressive strength. (**a**) Temperature, (**b**) Lithium slag content.

Figure 13 shows the cube compressive strength of LSC under different influencing factors. The effect of temperature on the normalized cube compressive strength was shown in Fig. 14a and b shows the influence trend of lithium slag content on the normalized cube compressive strength.

It showed that the cube compressive strength (*f*_cu_) decreased gradually as the temperature increased from 20 to 700 ℃. A simple linear regression is performed on the normalized cubic compressive strength values for all concrete specimens as shown in Fig. 14a. The linear regression equation of temperature and cube compressive strength is:

1 $$\frac{{f}_{cu,T}}{{f}_{cu,20}}=0.98632-0.00116T, {R}^{2}=0.98533$$

where T is the temperature, *f*_cu,T_ is the cubic compressive strength at temperature *T*, and *f*_cu,20_ is the cubic compressive strength at room temperature. It can be seen that the normalized cubic compressive strength value decreased with increasing temperature regardless of the lithium slag content, and the R^2^ value is 0.98533, indicating a strong statistical correlation between the two. Therefore, there was a linear relationship between the temperature and cubic compressive strength of LSC.

There was a nonlinear relationship between lithium slag content and cube compressive strength of LSC as shown in Fig. 14b. The water-binder ratio was 0.45, when the content of lithium slag in LSC increased gradually, *f*_cu_ showed a trend of rising first and then decreasing, and the *f*_cu_ of LSC was the largest when the content of LS was 20%.

At a temperature of 100 ℃, the *f*_cu_ of specimens dropped to 80% of the actual intensity, and the normalized values are all close to 0.8. After the 300 ℃ temperature, the *f*_cu_ dropped to between 60 and 70% of the actual strength. Compared with the normalized values at other temperatures, the normalized points at 300 ℃ are relatively dispersed. Compared with the temperature rose process of 100 ℃ to 300 ℃, the temperature rose from 300 to 700℃ made *f*_cu_ dropping faster. At 500 ℃ and 700 ℃, the *f*_cu_ of the specimens retained 40% and 20% of the actual intensity, respectively, and the normalized values of different specimens at these two temperatures are very close.

Compared with other temperatures, the *f*_cu_ of the specimen is preserved most completely at 100 ℃. The water inside the specimen gasified into water vapor at 100 ℃, but it could not escape completely, so that a large number of closed high-temperature and high-pressure air chambers were formed in the tiny pores inside the concrete specimen, which promoted the secondary progress of OPC hydration reaction^38^, the generated C-S-H filled the tiny gaps inside the specimen.

At 300 ℃, the normalized compressive strength of different LSC specimens was scattered, and the normalized compressive strength of concrete with 0% lithium slag content was slightly higher than that of other specimens. The normalized value of the specimen cooled by water spray was about 0.6, and that of the naturally cooled specimen is close to 0.7. The reason was that at 300 ℃, a large amount of free water and bound water in the concrete specimen had been lost as shown in Fig. 14a, resulting in a rapid weakening of the chemical bonding force of the C-S-H^34^. Due to the different proportions of lithium slag replacing OPC in the specimens, the degree of pozzolanic reaction of the concrete is different, and the loss of water is also different, so the strength loss was also different.

At 500 ℃ and 700 ℃, the normalized values are more concentrated, indicating that at high temperatures (500 ℃ and 700 ℃), the influence of lithium slag content and cooling method on *f*_cu_ is weakened. It had been pointed out in literature^34,39^ that a high temperature of 400 ℃ would dehydrate and decompose C-S-H, CH would dehydrate and decompose into calcium oxide (CaO) at an environment of 400 ℃ to 600 ℃, and when the temperature is between 600 and 800 ℃, calcium carbonate (CaCO_3_) would undergo a decarburization reaction. Therefore, after the specimen is heated at 500℃ and 700 ℃ for 3 h, the gel material inside the specimen is decomposed and dehydrated, and the strength decreased sharply.

Lithium slag content had a little effect on *f*_cu_. When the content of LS is 10%, *f*_cu_ has a slight increase. The *f*_cu_ of 20% lithium slag content increases the most. When the lithium slag content reaches 30%, the *f*_cu_ would not increase much, and even decrease. The reason is that LS contained a large amount of SiO_2_, adding an appropriate amount of LS in concrete would promote the full progress of the pozzolanic reaction, making the internal microstructure of the specimen more compact and the strength of the concrete higher. However, excessive LS would inhibit the generation of CH in OPC and weaken the pozzolanic reaction of LS^18^.

### Prism compressive strength

**Figure 15:**
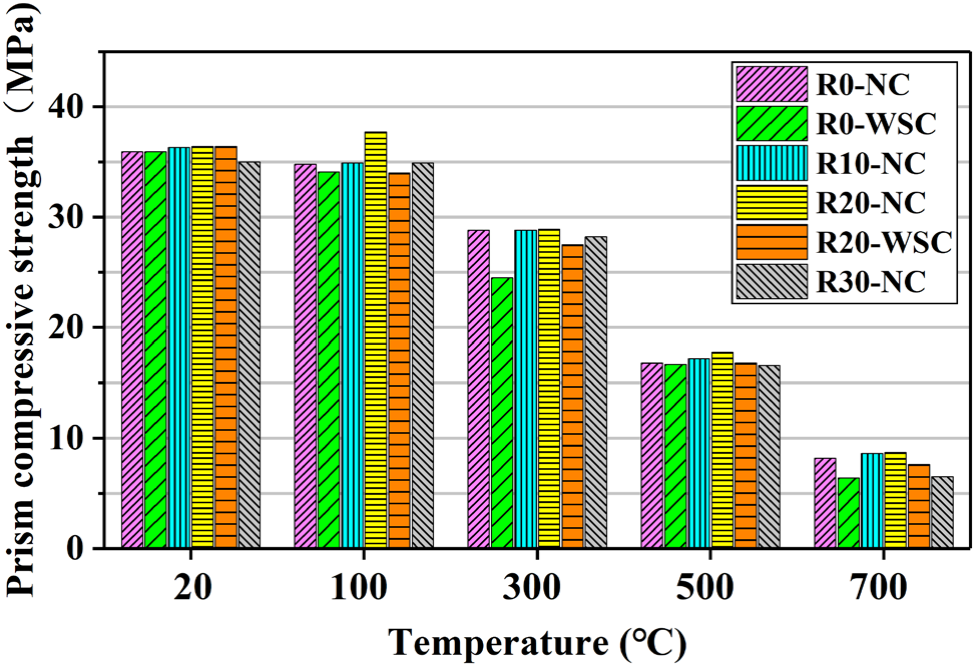
Prism compressive strength of LSC.

**Figure 16:**
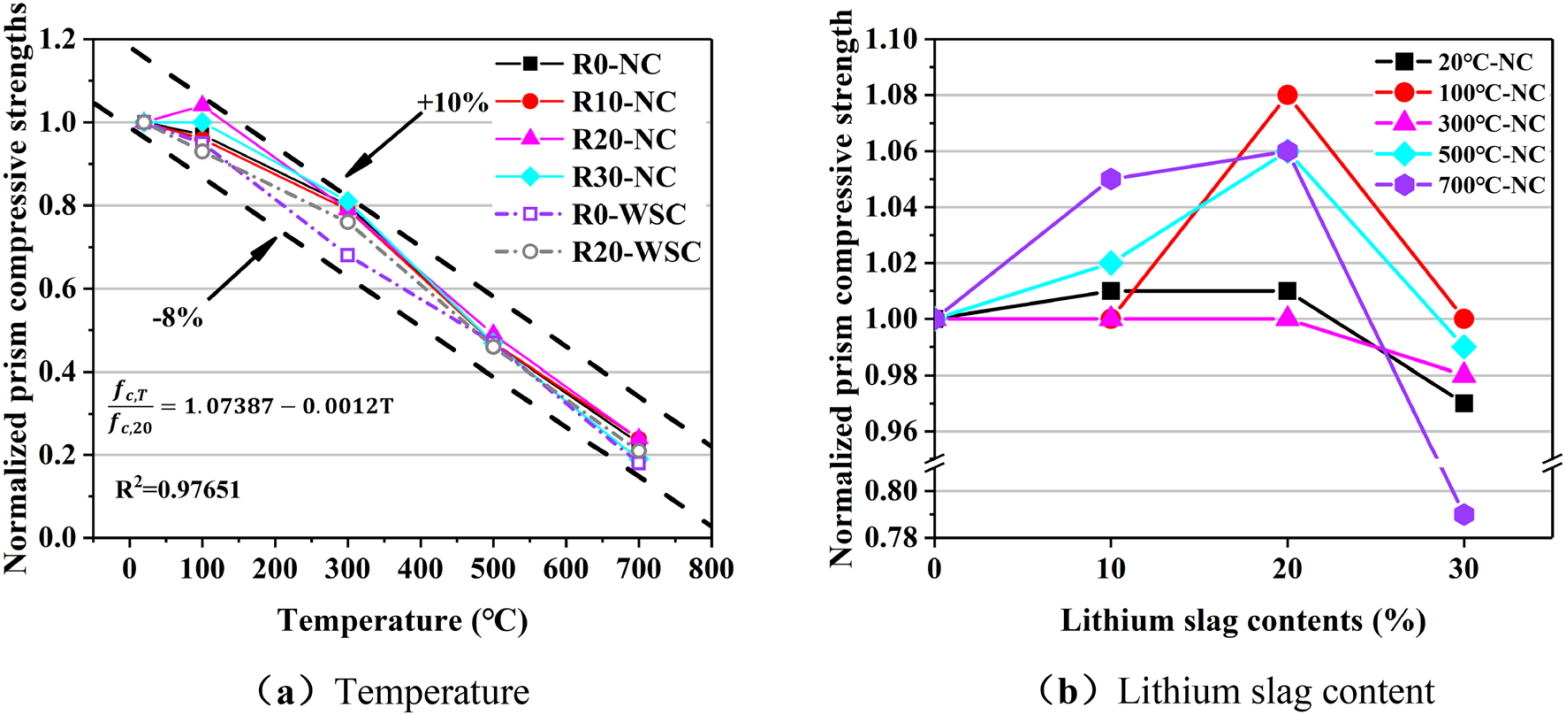
Normalized prism compressive strength. (**a**) Temperature, (**b**) Lithium slag content.

The prism compressive strength (*f*_c_) of the LCS is shown in Fig. 15. Figure 16a shows the influence of temperature on the normalized prism compressive strength, and the influence of lithium slag content on the normalized prism compressive strength is shown in Fig. 16b. Comparing the *f*_cu_ and *f*_c_, the effects of lithium slag content, cooling method and temperature on the two types of strength are very similar.

Linear regression analysis is performed on the normalized prism compressive strength values, as shown in Fig. 16a, and the Eq. (2) between temperature and *f*_c_ is obtained as follows:

2 $$\frac{{f}_{c,T}}{{f}_{c,20}}=1.07387-0.0012T, {R}^{2}=0.97651$$

where T is the temperature (℃), *f*_c,20_ is the prism compressive strength at room temperature, and* f*_c,T_ is the prism compressive strength when the temperature is T, ℃.

R^2^ = 0.97651, indicating a strong statistical correlation between temperature and *f*_c_. As the temperature increased, the *f*_c_ gradually decreased, and the relationship between the two was considered to be a linear change.

There is a nonlinear change between the content of lithium slag and *f*_c_ in LSC as shown in Fig. 16b. As the content of LS increased, the *f*_c_ increased first and then decreases. The *f*_c_ of the specimen with 20% lithium slag content was the largest.

Comparing the effect of temperature on *f*_cu_, the effect of temperature on *f*_c_ is slightly different as shown in Fig. 16a. At 100 ℃, the decrease in *f*_c_ is not significant, and the normalized values are all close to 1.0. Figure 15 and Fig. 16a showed that when the content of LS is 20%, *f*_c_ of the specimen is slightly improved at 100 ℃. The reason was explained in the previous section. On the other hand, at 100 ℃, the *f*_cu_ of the specimen still decreases, while the *f*_c_ of individual specimens not only does not decrease after exposure to 100 ℃, but increased slightly. The reason is that the volume of the specimen in the *f*_c_ experiment was three times that of the specimen in the *f*_cu_ experiment, so the secondary hydration reaction inside the specimen is more sufficient. The normalized values of the samples at 300 ℃ are between 0.7 and 0.8, and at 500 ℃ and 700 ℃, the normalized values are 0.5 and 0.2, respectively.

Comparing the normalized values of *f*_cu_ and *f*_c_, the normalized values of *f*_c_ at 100 ℃, 300 ℃ and 500 ℃ are greater than *f*_cu_, and at 700 ℃, the normalized values of *f*_cu_ and *f*_c_ are close. It was concluded that high-temperature would destroy the integrity of the concrete microstructure, allowing the C-S-H to be oxidatively decomposed. It completely decomposes the chemical components that mainly provide strength inside the concrete at 700 ℃, and the residual strength is only about one-fifth of the actual strength.

**Figure 17:**
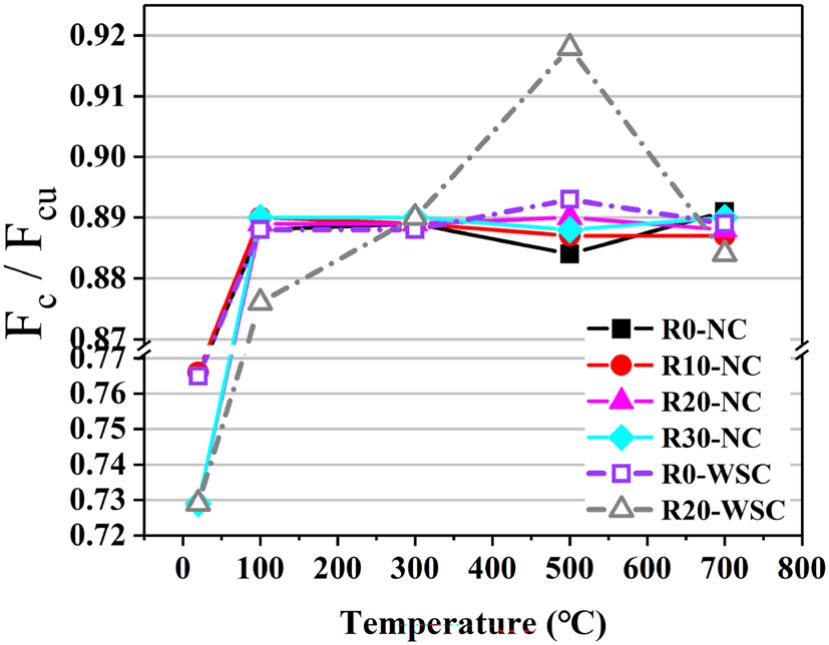
Intensity ratio.

Figure 17 shows the ratio of *f*_c_ to *f*_cu_ at different temperatures, called intensity ratio (*f*_c_/*f*_cu_), which is an index to measure the mechanical properties of concrete. The larger the intensity ratio, the better the performance of concrete. In this test, ordinary concrete was used as a control test, and the *f*_c_/*f*_cu_ was 0.76, which was consistent with the *f*_c_/*f*_cu_ = 0.76 of C50 and below regular concrete specified in the code. However, the intensity ratio of LSC specimens is all close to 0.89 or greater after high-temperature heating. Combined with the conclusions obtained in the previous article, it believes that this would cause less damage to the strength of concrete at 100 ℃, and if the concrete volume is large, it could also slightly increase the bearing capacity of concrete. This conclusion is similar to the conclusions of the two scholars^35,36^ mentioned in Sect. “Mass loss rate (ML)”, which proved the scientific nature of the conclusions drawn in this article.

### Flexural strength

**Figure 18:**
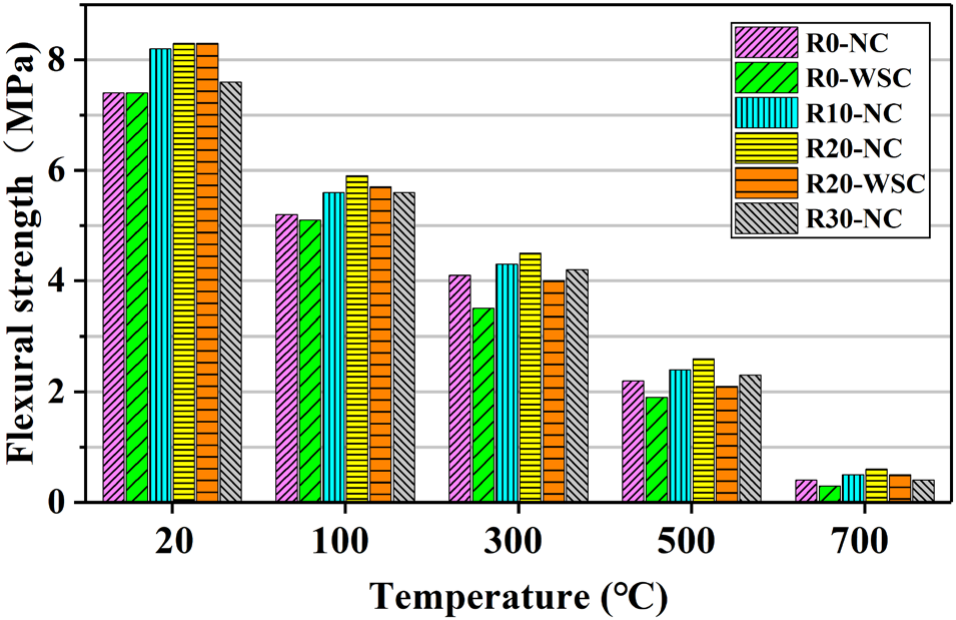
Flexural strength of LSC.

**Figure 19:**
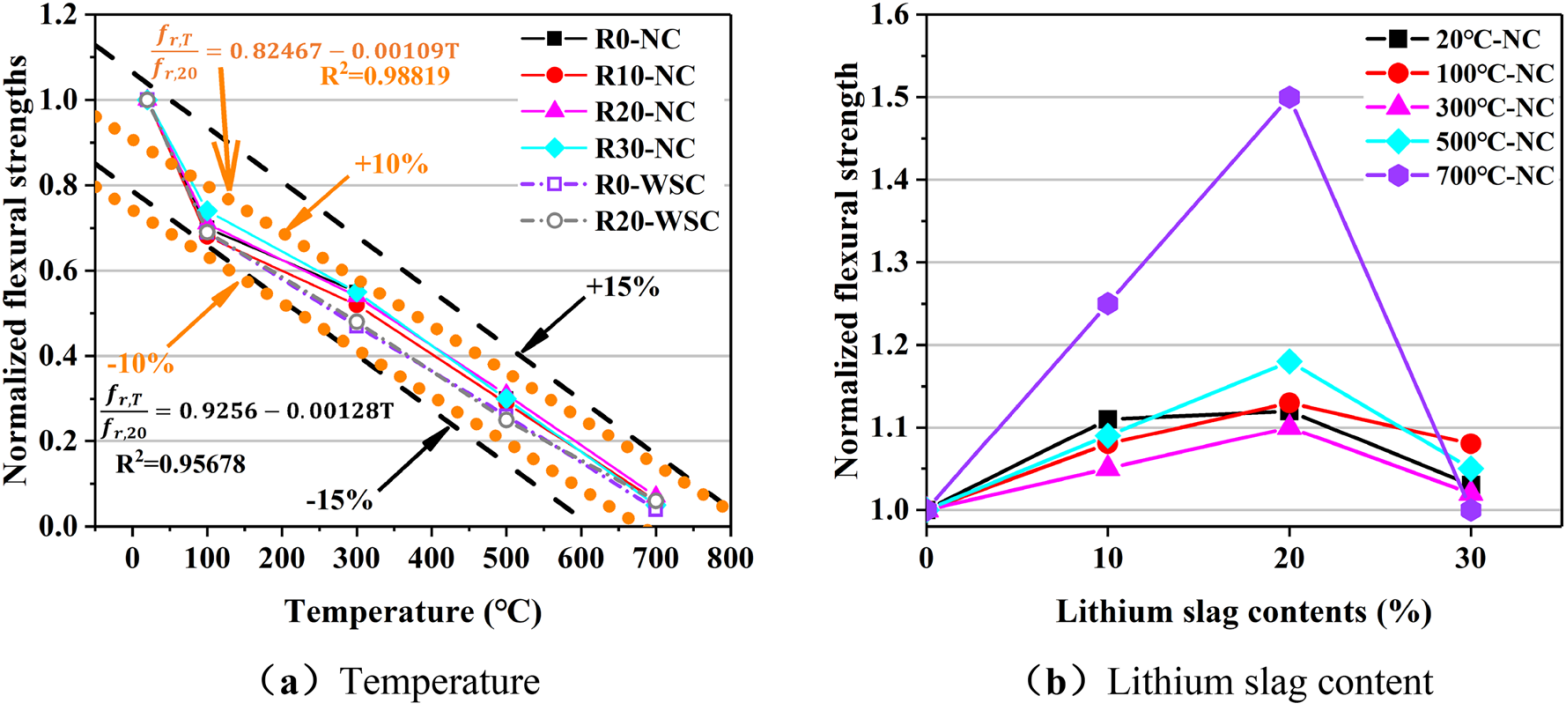
Normalized flexural strength. (**a**) Temperature, (**b**) Lithium slag content.

The flexural strength (*f*_r_) of LSC is shown in Fig. 18. The influence of temperature on the normalized value of *f*_r_ is shown in Fig. 19a, and Fig. 19b shows the influence of LS content on the normalized value of *f*_r_.

Figure 18 shows that high temperature (especially temperature higher than 300 ℃) damaged *f*_r_ more significantly. At 100 ℃, *f*_r_ of LSC is 70% of that at room temperature. At 300 ℃, *f*_r_ dropped by nearly 50%. At 500 ℃, *f*_r_ of the specimen is retained by 30%. At 700 ℃, *f*_r_ of the specimen is only 5% of that without high temperature. Therefore, it could be considered that *f*_r_ of LSC is completely lost in an environment of 700 ℃. The main reason for the gradual decrease of *f*_r_ to complete loss is the temperature. The main chemical components of LSC (C-S-H, CH, CaCO_3_) gradually hydrated and decomposed in different temperature ranges, so that the gel material between the coarse aggregates inside the LSC no longer worked.

Compared with *f*_cu_ and *f*_c_, LS have a greater influence on *f*_r_. The relationship between lithium slag content and *f*_r_ is nonlinear as shown in Fig. 19b. When the lithium slag content increases from 0 to 10%, *f*_r_ increases by about 5%. When the lithium slag content is 20%, *f*_r_ increases the most. When the LS content reaches 30%, *f*_r_ drops to the level when the LS content was 10%.

In this paper, a linear regression analysis was performed on the normalized value of *f*_r_, as shown in Fig. 19a. Considering the normalized value at room temperature, the relationship between temperature and *f*_r_ is obtained as shown in Eq. (3):

3 $$\frac{{f}_{r,T}}{{f}_{r,20}}=1.07387-0.0012T, {R}^{2}=0.95678$$

When the normalized value at room temperature is not considered, the relationship between the two is shown in Eq. (4):

4 $$\frac{{f}_{r,T}}{{f}_{r,20}}=0.82467-0.00109T, {R}^{2}=0.98819$$

where T is the temperature, *f*_r,20_ is the flexural strength at room temperature, and *f*_r,T_ is the flexural strength when the temperature is T, ℃. Comparing Eq. (3) and Eq. (4), without considering the normalized value of 1, the dispersion of temperature and *f*_r_ is smaller, R^2^ = 0.98816, and the statistical correlation between the two is stronger. It can be concluded that the relationship between temperature and *f*_r_ varied linearly.

Naveen et al.^23^ pointed out that at 800 ℃, all concrete specimens failed to undergo flexural strength tests. Putting the test value at 800 ℃ into the Eqs. (3) and (4), *f*_r_ is negative, which is the same as the conclusion in the literature. It is proved that the formula obtained in this paper was practical. Therefore, it is recommended that when buildings with flexural strength requirements are exposed to fire and high-temperature environments, the surface of concrete components should be treated with fire prevention and heat insula.

### Effect of cooling method

In this test, the effect of WSC on *f*_cu_ (Fig. 13), *f*_u_ (Fig. 15) and *f*_r_ (Fig. 18) of the concrete specimens was not obvious, and the strength was only slightly lower than that of the naturally cooled specimens. This phenomenon was quite different from the conclusions drawn by other scholars^29,32,34^. This was because the duration of water spray cooling for the specimen in this experiment was 3–5 min, which was the same as one of the water spray cooling methods in literature^40^. In that document, cooling by spraying water for 5 min had little effect on the strength of concrete specimens, which was similar to the situation in this experiment. Therefore, the effect of WSC time on the strength of LSC after high temperature remains to be studied.

## Conclusion

1. The mass loss rate (ML) of LSC increases rapidly as the heating temperature increases. The ML of LSC at 100 ℃ is about 1%. At a temperature of 300 °C, the ML reaches 4–6%. Temperature in the interval of 300–700℃, the temperature and mass loss rate tend to increase linearly, and the rate of increase is less than 100–300℃.
2. At a temperature of 100 ℃, the *f*_cu_ and *f*_r_ of LSC decrease by 20% and 30%, respectively. However, the *f*_c_ of specimens with 20% lithium slag substitution rate increased by 8%. At a temperature of 700 °C, the *f*_cu_ and *f*_c_ of LSC decreased by 80%, and the flexural strength was almost lost. Therefore, more attention must be paid to the LSC specimens that are to have fr after high temperatures in engineering.
3. The W/B of LSC was 0.45. When the LS replacement rate increased from 0 to 30%, the *f*_cu_, *f*_c_ and *f*_r_ of LSC all increased first and then decreased. The LS replacement rate with the greatest effect on strength improvement was 20%, with *f*_cu_ increased by 5–8%, *f*_c_ increased by 6–8% and *f*_r_ increased by 10–20%. At a temperature of 700 ℃, the *f*_r_ of LSC with a 20% LS replacement rate was 50% higher than that of ordinary concrete.
4. The intensity ratio (*f*_c_/*f*_cu_) was greater after high temperature than that at the normal temperature. When the temperature was in the range of 100–700 ℃, the intensity ratios of all specimens were greater than 0.88. Considering that the *f*_cu_ and *f*_r_ of concrete were reduced by nearly 20% at 100 ℃.

## Recommendations

Considering the time required for fire extinguishing at the fire scene, the effect of WSC duration on the mechanical properties of lithium slag concrete can be further studied in the future. Because WSC will reduce the mechanical properties of concrete, the WSC time was short in this test, and the effect of water spray duration on the strength of the specimen was not studied.

## Data Availability

All data generated or analysed during this study are included in this published article.

## Abbreviations

LS: Lithium slag
OPC: Ordinary Portland cement
SCM: Supplementary cementitious material
LSC: Lithium slag concrete
NC: Natural cooling
WSC: Water spray cooling
XRD: X-ray diffraction
GB/T: Chinese standard
JCJ: Chinese standard
ML: Mass loss rate
C-S–H: Hydrated calcium silicate gel
CH: Calcium hydroxide
CaO: Calcium oxide
CaCO_3_: Calcium carbonate
SO_3_: Sulfur trioxide
SiO_2_: Silicon dioxide
Al_2_O_3_: Aluminum oxide
Fe_2_O_3_: Ferric oxide
CaO: Calcium oxide
MgO: Magnesium oxide
W/B: Water-binder ratio
*f*_cu_: Cube compressive strength
*f*_c_: Prism compressive strength
*f*_c_/*f*_cu_: Intensity ratio
*f*_r_: Flexural strength
